# Searching for the forest ghosts: group counts and polyspecific associations of the endangered *Rungwecebus kipunji* in the Udzungwa Mountains, Tanzania

**DOI:** 10.1007/s10329-026-01250-7

**Published:** 2026-03-06

**Authors:** Claudia Barelli, Trevor Jones, Richard Laizzer, Steven Shinyambala, Athumani Mndeme, Francesco Rovero

**Affiliations:** 1 Department of Biology, University of Florence, Sesto Fiorentino, Italy; 2 Udzungwa Landscape Strategy, Box 2494, Iringa, Tanzania; 3 Southern Tanzania Elephant Program (STEP), Wilolesi, Iringa, Tanzania; 4 Udzungwa Ecological Monitoring Centre (UEMC), Udzungwa Mountains National Park, Mang’ula, Tanzania; 5 MUSE, Science Museum of Trento, Trento, Italy

**Keywords:** Line transects, Mixed-species interactions, Primate census, Peter’s Angola colobus, Tanzania Sykes’ monkeys, Udzungwa red colobus

## Abstract

The IUCN-Endangered kipunji (*Rungwecebus kipunji*) is one of Africa’s rarest primates, restricted to two isolated populations in Tanzania. The Udzungwa population is enigmatic, confined to a small area within a large, old-growth forest and consists of fewer than 100 individuals. Apart from baseline data from 2006, ecological knowledge remains scant. We present new data from three standardized sweep censuses conducted between 2013 and 2024 in Ndundulu forest along 42–45 linear transects of 2 km in length. Per census, only 4 to 6 kipunji groups were recorded, within a range of 10.42 km^2^ which includes opportunistic sightings. Encounter rate averaged 0.04 groups/km, 8 to 12 times less than for the three other diurnal primates co-occurring there (*Colobus angolensis palliatus, Cercopithecus mitis monoides*, *Piliocolobus gordonorum*). Mean (SD) minimum group size was 17 ± 2 individuals, giving a crude and minimum abundance estimation per census that varied from 51 ± 6 to 102 ± 12 individuals. Despite occurring in high-quality, undisturbed and well-protected habitat, kipunji in Udzungwa show puzzlingly low density and a small range. Kipunji were observed in association with the other three diurnal primates in 43.8% of encounters, mainly forming dyads with Tanzania Sykes’ monkeys (42.9%) and, less frequently, with Udzungwa red colobus (14.3%). However, Sykes’ monkeys associated the least and were the only species whose relative abundance increased, suggesting niche dominance that may particularly affect the kipunji due to shared dietary preferences. We warn that kipunji persistence in Udzungwa is at risk and urge intensified monitoring and continued protection of the Kilombero Nature Forest Reserve.

## Introduction

Knowledge on distribution and abundance of endangered species is essential to assess population dynamics, ecological adaptations, and the effectiveness of conservation measures (Kleiman et al. 2001; Magurran et al. 2010). Endemic and threatened taxa, with their restricted distributions and vulnerability to environmental change, serve as critical indicators of ecosystem integrity and conservation success, especially in rapidly changing tropical ecosystems (Pillay et al. 2024). In this context, non-human primates play a crucial ecological role, given their high conservation relevance and their influence on tropical biodiversity, ecosystem processes, and forest regeneration (Estrada et al. 2017, 2018). Within African forests, the IUCN-Endangered kipunji, *Rungwecebus kipunji*, stands out as both a flagship species for the forests in Tanzania where it is found and an indicator of their ecological health (Davenport et al. 2013; Davenport 2019). First documented by scientists in the Southern Highlands and Udzungwa Mountains in 2003 and 2004, respectively (Davenport et al. 2006; Jones et al. 2005), the kipunji remains one of Africa’s rarest and most enigmatic primates. Its distribution is confined to two isolated populations: one in the Southern Highlands (Mount Rungwe and Livingstone; area of occupancy 1,079 ha; elevation 1,400–2,960 m; Bracebridge et al. 2012; Davenport et al. 2008), and one in the western Udzungwa Mountains (Ndundulu forest; area of occupancy 199 ha; elevation 1,300–1,750 m; Davenport et al. 2006, 2008; Jones et al. 2005), which are separated by 370 km of mainly non-forested and agricultural landscapes, with no evidence of connectivity in the recent past (minimum 100–150 years).

The Udzungwa population occurs within the Kilombero Nature Forest Reserve (KNFR), one of Tanzania’s most ecologically significant reserves (Davenport et al. 2013; Ract et al. 2024), encompassing extensive tracts of pristine rainforest with exceptional levels of endemism (Dinesen et al. 2001; Rovero et al. 2008). However, despite the high ecological value of this reserve, raw densities of the kipunji there were estimated to be more than three times lower than in the mostly secondary forests where the Southern Highland population occurs (Davenport et al. 2008). Reasons for these lower densities in Udzungwa are unknown, but inbreeding depression, disease susceptibility and historical hunting pressure by local communities cannot be ruled out. Moreover, kipunji in the KFNR is reported to be confined to only about 25 km^2^ (Davenport 2019) within the 231 km^2^ of the Ndundulu forest (hence less than 10% of the available habitat). According to the only published account reporting population estimates, it may represent just 7% (ca. 75 individuals) of the total known population, with the vast majority (93%, 1,042 individuals) residing in the Southern Highlands (Davenport et al. 2022; Marshall et al. 2015).

Twenty years after its discovery, we still do not understand the reasons for the extremely restricted range and low density of kipunji in the Udzungwa Mountains. First, although anthropogenic habitat degradation and bushmeat hunting threatens the primates in some of the less well managed forests in the Udzungwa Mountains (Barelli et al. 2023; Rovero et al. 2012), quantitative habitat assessments in Ndundulu forest show that the forest portion currently occupied by the kipunji is of high quality in terms of both arboreal structure and tree diversity, comparable to or even exceeding that of nearby forests (Marshall et al. 2015). This suggests that forest degradation and other forms of anthropogenic disturbance cannot currently explain the low density and restricted distribution of kipunji in the Udzungwa Mountains. Second, genetic differences between the two populations may provide part of the explanation; indeed, the Southern Highlands one carries *Papio*-derived mtDNA, while the Udzungwa population retains the only *Rungwecebus* lineage, reflecting long-term isolation and past introgression (Zinner et al. 2018). Although morphologically similar, these genetic differences could affect population fitness — for example through reduced genetic diversity or adaptive potential — and, together with factors such as historical demographic bottlenecks, social constraints, or other ecological pressures, may limit population size and survival of kipunji in the Udzungwa Mountains (Bracebridge et al. 2011; Davenport et al. 2008; Marshall et al. 2010).

Third, competition with other papionids (i.e., Sanje mangabey *Cercocebus sanjei* and yellow baboon *Papio cynocephalus*), which are absent from the Southern Highlands, may have contributed to the current confinement of kipunji in KNFR, given that both of those species do not occur in that forest (Bracebridge et al. 2011). The presence of other sympatric but ecologically distinct arboreal and diurnal primates, with which kipunji regularly associate (Davenport and Butynski 2013), may also help explain the species’ persistence ability. In the Southern Highlands, kipunji were observed interacting with both Peter’s Angola colobus (*Colobus angolensis palliatus*) and Tanzania Sykes’ monkey (*Cercopithecus mitis monoides*), during both early mornings and late afternoons, and the three species were often observed to sleep in neighbouring trees (Devenport et al. 2006). In the Udzungwa Mountains, such interactions also include the endemic Udzungwa red colobus (*Piliocolobus gordonorum*) (Davenport and Butynski 2013) although systematic observations on these associations have not been reported for either of the populations. As in many diurnal primates, such mixed-species interactions (hereafter referred to as polyspecific associations) can strongly influence habitat use, foraging strategies, and anti-predatory behaviour through shared vigilance, increased foraging efficiency, or social facilitation (Chapman and Chapman 1996, 2000; Cords 1990a,b; Gautier-Hion and Gautier 1983; Waser 1980). Abundance and distribution data on the Udzungwa population of the kipunji have only been published from a series of baseline counts conducted in 2006 (Davenport et al. 2008). Here, we report on three subsequent and standardized sweep census surveys, in addition to social structure and polyspecific association data. Therefore, we aimed to contribute knowledge towards understanding the critical and enigmatic status of this IUCN-Endangered primate in the Udzungwa mountains.

## Material and methods

### Study area and animal populations

**Fig. 1:**
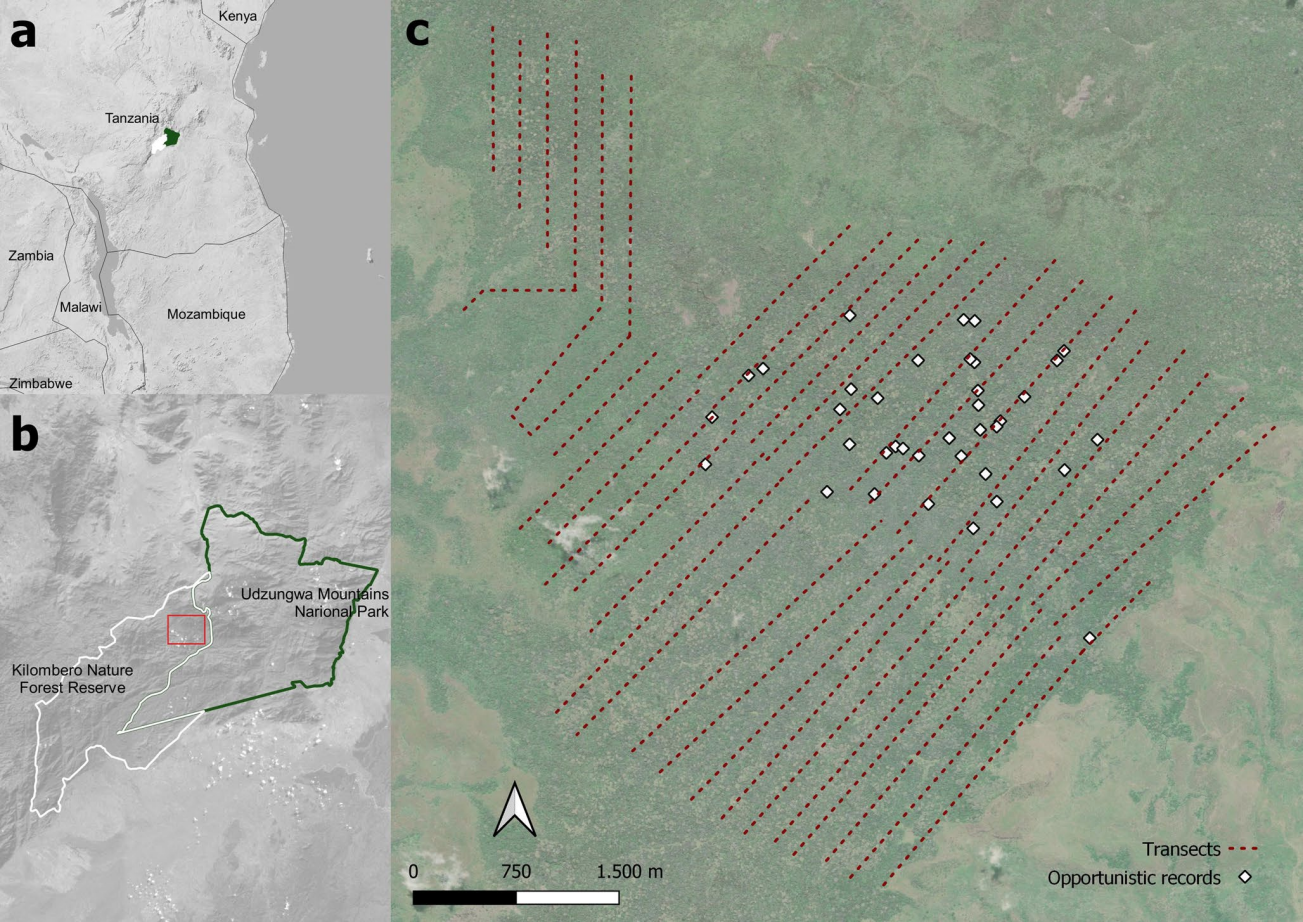
Maps of the Udzungwa Mountains, kipunji (*Rungwecebus kipunji*) distribution and line transects. The maps show (**a**) the location of the Udzungwa Mountains of south-central Tanzania, (**b**) the Kilombero Nature Forest Reserve (KNFR) within the Udzungwa Mountains where kipunji has been recorded, and (**c**) all transects used for primate counts shown as dotted lines across the Ndundulu forest within the KNFR. The boundaries of the Udzungwa Mountains National Park in Tanzania are shown with green continuous lines while those of the KNFR in white (panels (**a**) and (**b**)). The background layer of panel (**c**) represents a Digital Elevation Model (where darker shades indicate lower elevations), while squared white symbols represent opportunistic observations of kipunji before systematic transect surveys commenced

Ndundulu forest (36°30′E, 7°45′S) is located in the Udzungwa Mountains of south-central Tanzania and is part of the larger KNFR, that extends over 1,345 km^2^. The West Kilombero Scarp Forest Reserve (WKSFR), established in 1957, originally encompassed the entire Ndundulu–Luhomero massif, a large tract of contiguous forest. In 1992, the gazettement of the Udzungwa Mountains National Park (UMNP) incorporated approximately 75% of this area, leaving the remaining portion—including Ndundulu forest—within the reduced WKSFR (Fig. 1a, b). In 2007, this reduced WKSFR was upgraded to Nature Forest Reserve status (Marshall et al. 2007), which meant stricter protection measures and law enforcement. Protection efforts for this priority forest for biodiversity conservation have been supported by the Southern Tanzania Elephant Program (STEP) since 2016, and patrols in collaboration with the Tanzania Forest Services (TFS), Tanzania National Parks (TANAPA) and local communities have expanded in frequency and coverage since 2024 under the Udzungwa Landscape Strategy.

The vegetation of Ndundulu forest is dominated by primary mature submontane forest between 1,300 and 2,000 m a.s.l., transitioning into upper montane forest (Lovett and Pócs 1993) at higher elevations. Notably, kipunji is found almost exclusively within the southern, lower elevation portion of Ndundulu, a tract of intact, moist submontane forest (~ 1,300–1,750 m a.s.l.), while in the Southern Highlands kipunji occurs at ~ 1,750–2,450 m a.s.l. (Davenport 2019). Ndundulu is indeed recognised as one of the most biodiverse forests in both the Udzungwa Mountains and the broader Eastern Arc range (Dinesen et al. 2001). Species new to science discovered within this forest include the Udzungwa forest-partridge *Xenoperdix udzungwensis* (Dinesen et al. 1994), Phillips’ shrew *Congosorex phillipsorum* (Stanley et al. 2005), and the grey-faced sengi *Rhynchocyon udzungwensis* (Rovero et al. 2008).

We focused on the kipunji and the three other diurnal species that co-occur in Ndundulu, whose differences and similarities in social organization, habitat use, and diet are likely key factors shaping their regular interspecific co-occurrence and interaction that we report: Peter’s Angola colobus, Tanzania Sykes’ monkey, and the endemic Udzungwa red colobus. Little is known on the ecology of the Udzungwa population of kipunji, as most data come from the Southern Highlands one. The species is strictly forest-dependent and arboreal, though in the Southern Highlands individuals are occasionally found on the ground and outside the forest to exploit crops such as banana and maize (Davenport 2005; Davenport et al. 2006). In the Southern Highlands, home ranges are reported to average 3.06 km^2^ and are generally non-territorial (De Luca et al. 2010). Adult males have an estimated head–body length of 85–90 cm and weigh approximately 10–16 kg (Davenport et al. 2006). Their diet in the Southern Highlands is reported to include fruits, leaves, bark, and moss, and shifts seasonally, with a higher leaf consumption during the dry season and increased fruit intake during the rainy season (Bracebridge et al. 2012; Davenport et al. 2010). Data on diet have been collected opportunistically in Udzungwa, with approximately 20 species of trees identified and an apparent preference for fruits and seeds, but also including young leaves, buds and lichen (TJ, unpubl.). Kipunji are reported to form multi-male/multi-female social groups with 15–25 individuals (Davenport et al. 2008). Regarding the other diurnal primates, Peter’s Angola colobus typically live in one-male/multi-female groups, ranging from 2 to 14 individuals per group (Rovero et al. 2009), are largely arboreal, and feed primarily on mature leaves (Bocian 1997; Fimbel et al. 2001). Adult males are slightly larger than females, with head–body lengths of approximately 71 cm and body weights of about 9.7 kg (range 8–13 kg; Cunneyworth et al. 2020). Tanzania Sykes’ monkey also form one-male/multi-female groups (2–22 individuals; Rovero et al. 2009), use both lower and upper canopy strata and feed mainly on fruits (Rovero et al. 2009). Considered medium-sized primates, male Tanzania Sykes’ monkeys typically weigh 6–9 kg, with females considerably smaller (3–6 kg), and have a head-to-body length ranging from 50 to 70 cm. (Groves 2001). Like kipunji, Udzungwa red colobus also occur in multi-male/multi-female groups (3–84 individuals; Rovero et al. 2009), are predominantly arboreal, and prefer young leaves, with males being slightly larger than females, weighing 9–13 kg versus 7–9 kg in females, and having head-body lengths of 46–70 cm (Struhsaker 2010).

## Data collection

We used sweep censuses to count primate groups, whereby we systematically walked parallel transects in a coordinated manner to detect and record every group encountered. Transects were largely consistent in their placement across survey years. This method, used in the baseline study conducted in March 2006 (Davenport et al. 2008) ensured the comprehensive coverage of the study area. Censuses were repeated in 2013, 2016 and 2024, during the dry season (i.e. within August-October) for easier detection of animals as March usually falls within the rainy season. Sampling uniformly covered the largest-recorded kipunji’s known range estimated at 25 km^2^ (Davenport 2019), which is in the southern and western part of Ndundulu forest. Censuses consisted of three teams, each composed of two trained field assistants, who walked between 42 and 45 pre-planned linear transects, each approximately 2 km long and spaced 100–300 m apart (Fig. 1c), with three adjacent transects surveyed simultaneously each census session. Teams used handheld GPS units, with the prerecorded transect routes, to walk the line with precision. The spacing was designed to minimize both double counting and the risk of missing groups in between lines. Each day, one census was conducted in the morning and one in the afternoon, hence six transects were walked each day and the whole census was usually completed in 7–8 days. Observers moved to a different, adjacent area each day, ensuring that no transect was surveyed more than once. For each diurnal primate group encountered, we recorded location, elevation, an estimation of minimum group size, and polyspecific associations. Data on group size and especially composition (age and sex) were collected systematically on all censuses, however it is important to note that due to animal elusiveness and dense forest habitat, counts are not to be considered complete, and attribution to sex and age classes was only possible for a fraction of individuals. The largest sample of group size and composition was collected in 2024, therefore the results presented here are primarily based on observations from that year. Observers were thoroughly trained prior to data collection, by walking trial transects to minimize potential differences in animal detection capability. Two primary observers participated across all four study periods, ensuring continuity and comparability of the observations.

## Data analysis

The low sample sizes did not allow us to use statistical testing, including inferential density estimation through distance sampling. We calculated mean values and standard deviations to summarize group (defined as ≥ 2 individuals) composition, including the number of individuals and age-sex structure and the social groups’ encounter rates per each species (i.e., number of groups seen divided by distance walked). Given that the low visibility and elusiveness of animals limited the ability to count individuals accurately during censuses, we used the average group count obtained from the 2024 census and multiplied it by the number of groups seen per census to compute a crude estimation of individual abundance from across all censuses. We identified polyspecific associations as the co-occurrence of different species within a 50 m diameter sphere (Mitani 1991), typically implying co-presence of individuals from two or more species in the same or adjacent trees. We pooled polyspecific association data from all years to analyse their frequency on all sightings and species composition.

## Results

### Survey effort, distribution and abundance

**Fig. 2:**
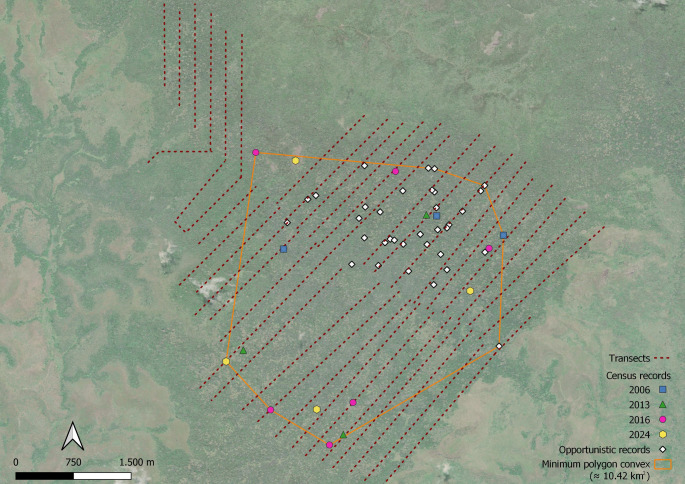
Records of kipunji (*Rungwecebus kipunji*) group observations across years. Each coloured dot represents the geographic coordinates of a record from a specific year during transect surveys. White squares indicate opportunistic observations made between 2004 and 2006, prior to the establishment of standardized transects. The orange line represents the minimum convex polygon (MCP) encompassing all records

Across the study area, transect lengths and survey effort have been broadly comparable since the baseline census in 2006, when 61.23 km were walked over 132 survey hours. In 2024, a total of 86.58 km were walked across 43 transects (range: 0.71–3.84 km per transect) over ten consecutive days, for a total effort of 131 h (Fig. 2), while 91.6 km were walked in 2013 and 93.5 km in 2016 (Table 1). Kipunji groups were detected along 4 of the 43 transects in 2024, 6 in 2016 and 3 in 2013 and were concentrated in the southern and western sections of Ndundulu forest. This distribution largely overlaps with that recorded in 2006 from both the sweep census (that recorded 4 groups) and several opportunistic sightings that were recorded by TJ and AM during collection of feeding and behavioural data from unhabituated groups in the same year (Table 1, Fig. 2). The minimum convex polygon (MCP), calculated using all sightings, reveals a minimum extent of occurrence of 10.42 km^2^ (Fig. 2). In 2024, a minimum of 55 individuals were counted from the 4 group sightings; when applying the average of 17 ± 2.00 (SD) individuals per group (details below) to all censuses, the estimated minimum abundance varies from 51 (45—57) to 102 (90—114) individuals per census (68 in 2024; Table 1).

**Table 1:** Survey effort and Kipunji group detections across different census years

Year	Months	Duration (hrs/mins)	Total length walked (km)	Mean km walked (min–max)	Total transects walked	Observed kipunji groups	Estimated abundance^a^
2006	Mar 8th – 14th	132:31	61.23	2.2 (0.9—3.2)	42	3	51
2013	Aug 8th −16th	112:15	91.6	2.07 (0.9—3.3)	42	3	51
2016	Sep 30th—Oct 9th	122:04	93.5	2.01 (0.71—3.84)	45	6	102
2024	Aug 31st—Sep 9th	131:18	86.58	2.2 (0.9—3.2)	43	4	68

^a^Abundance was calculated by multiplying the number of groups observed across years by the average number of individuals found in each social group according to the 2024 survey

**Fig. 3:**
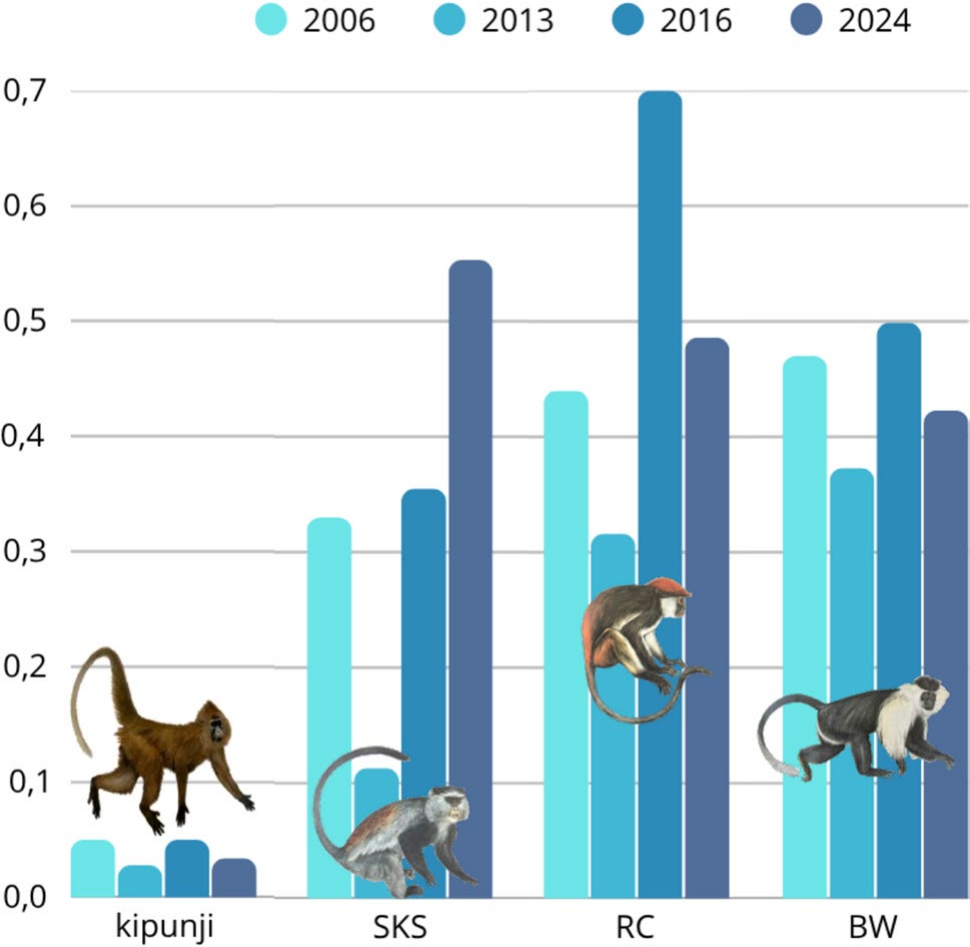
Encounter rates (number of groups observed per km walked) of primates across survey years. Mean observed counts (group/km) in the Ndundulu forest within the Udzungwa Mountains, Tanzania, are shown for the kipunji (*Rungwecebus kipunji*), Tanzania Sykes’ monkey Sykes (*Cercopithecus mitis monoides*, SKS), Udzungwa red colobus (*Piliocolobus gordonorum*, RC), and Peters’ Angola colobus (*Colobus angolensis palliates*, BW) across four distinct colour-coded years. Primate drawings by Jonathan Kingdon (Butynski et al. 2013)

Udzungwa red colobus were recorded on 19 transects in 2013, 28 in 2016, and 26 in 2024, while this information for the 2006 baseline survey is lacking. Observations in 2024 were mostly concentrated at mid- to high-elevation sites (1,319–1,859 m a.s.l.). Tanzania Sykes’ monkeys were detected on 11 transects in 2013, 21 in 2016, and 26 in 2024, showing a clear increase in spatial occurrence over time. Peter’s Angola colobus was observed on 24 transects in 2013, 21 in 2016, and 25 in 2024 between 1,362 and 1,856 m a.s.l.. A total of 107 primate groups were recorded in 2024, including 4 kipunji, 32 Udzungwa red colobus, 38 Tanzania Sykes’ monkeys, and 33 Peter’s Angola colobus. These values broadly align with previous censuses (Table 2). Encounter rates were 0.03 (SD = 0.11) for kipunji, 0.49 (SD = 0.45) for Udzungwa red colobus, 0.55 (SD = 0.72) for Tanzania Sykes’ monkeys, and 0.42 (SD = 0.42) for Peter’s Angola colobus. Encounter rates varied across years for all species with no clear pattern of changes detectable; however, kipunji remained consistently rare, while Tanzania Sykes’ monkey was the only species for which a relative increase since 2013 is apparent (Table 2, Fig. 3).

**Table 2:** Encounter rates (mean ± SD; number of groups per km walked) and, in brackets, the total number of groups observed during the survey for all four diurnal primate species across years. For the baseline year (2006), only mean values are shown (standard deviation not applicable)

Year	Kipunji	RC	SKS	BW
2006	0.05 ± n.a. (3)	0.44 ± n.a. (27)	0.33 ± n.a. (20)	0.47 ± n.a. (29)
2013	0.03 ± 0.10 (3)	0.32 ± 0.40 (28)	0.11 ± 0.19 (11)	0.37 ± 0.39 (32)
2016	0.05 ± 0.13 (6)	0.70 ± 0.85 (42)	0.36 ± 0.64 (14)	0.5 ± 0.83 (28)
2024	0.03 ± 0.11 (4)	0.49 ± 0.45 (32)	0.55 ± 0.72 (38)	0.42 ± 0.42 (33)

*RC*: Udzungwa red colobus (*Piliocolobus gordonorum*); *SKS*: Tanzania Sykes’ monkey Sykes (*Cercopithecus mitis monoides*); *BW*: (*Colobus angolensis palliates*); *N.A*.: not applicable

## Group composition

Due to the elusiveness of non-habituated animals and limited visibility in the dense canopy, individuals of undetermined age or sex accounted for a relatively large fraction of observations (32.8% in Peter’s Angola colobus to 44.2% in Udzungwa red colobus), therefore the age–sex proportions that we report hereafter are to be considered partial and approximate (Table 3). From the 2024 survey, group sizes varied considerably among species, ranging from small one-male/multi-female units in Peter’s Angola colobus and Tanzania Sykes’ monkey to larger multi-male/multi-female aggregations in kipunji and Udzungwa red colobus. Mean group size was highest in Udzungwa red colobus (mean ± SD = 19.75 ± 9.78 individuals) and lowest in Tanzania Sykes’ monkey (4.74 ± 2.20). The minimum counts for Kipunji groups averaged 17.00 ± 2.00 (SD) individuals, consistent with the literature and previous estimations, while Peter’s Angola colobus groups showed intermediate values (7.07 ± 3.34). The observed age–sex compositions appeared consistent with the patterns reported for these species in other areas. In all cases, adult females represented the largest proportion of individuals, except for Tanzania Sykes’ monkeys, in which the proportion of adult males (25.2%) was closer to that of females (20.3%) and therefore higher than expected). The proportion of subadults and juveniles ranged from 4.8% in Tanzania Sykes’ monkey to 12.7% in kipunji, whereas infants accounted for 7.3% to 18.3% of group members, being most frequent in Udzungwa red colobus and Tanzania Sykes’ monkey and lowest in kipunji.

**Table 3:** Summary of mean group size (± SD), minimum group composition observed, and percentage of sex and age class per species for 2024 survey

Species	Group size (mean ± SD)	Range	% AM	% AF	% S/J	% I	% Unk	# Groups
kipunji	17.00 ± 2.00	14–18	14.55	23.64	12.73	7.27	41.82	4
RC	19.75 ± 9.78	4–38	5.31	23.29	8.90	18.32	44.18	32
SKS	4.74 ± 2.20	2–10	25.20	20.30	4.80	15.45	34.10	42
BW	7.07 ± 3.34	2–16	16.76	24.28	9.25	14.45	35.26	33

*RC*: Udzungwa red colobus (*Piliocolobus gordonorum*); *SKS*: Tanzania Sykes’ monkey Sykes (*Cercopithecus mitis monoides*); *BW*: (*Colobus angolensis palliates*); *AM*: adult male; *AF*: adult female; *S/J*: subadult/juvenile; *I*: infant; Unk: individuals not classified by age or sex class

## Polyspecific associations

**Fig. 4:**
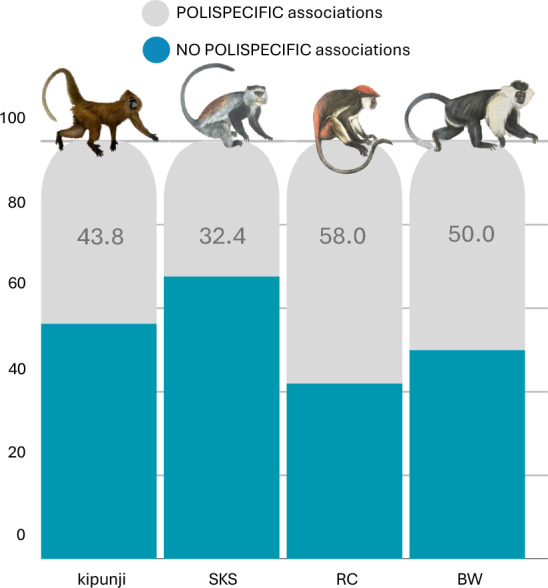
Polyspecific associations across Udzungwa primates. Percentage of polyspecific associations for the kipunji (*Rungwecebus kipunji*), Tanzania Sykes’ monkey Sykes (*Cercopithecus mitis monoides*, SKS), Udzungwa red colobus (*Piliocolobus gordonorum*, RC), and Peters’ Angola colobus (*Colobus angolensis palliates*, BW). Bar plots show the proportion of observations, combining data for all four survey years, in which each species was recorded in association with other primate species (“POLYSPECIFIC associations”, shown in gray) versus those observed alone (“NO POLYSPECIFIC associations”, shown in light blue). Primate drawings by Jonathan Kingdon (Butynski et al. 2013)

**Fig. 5:**
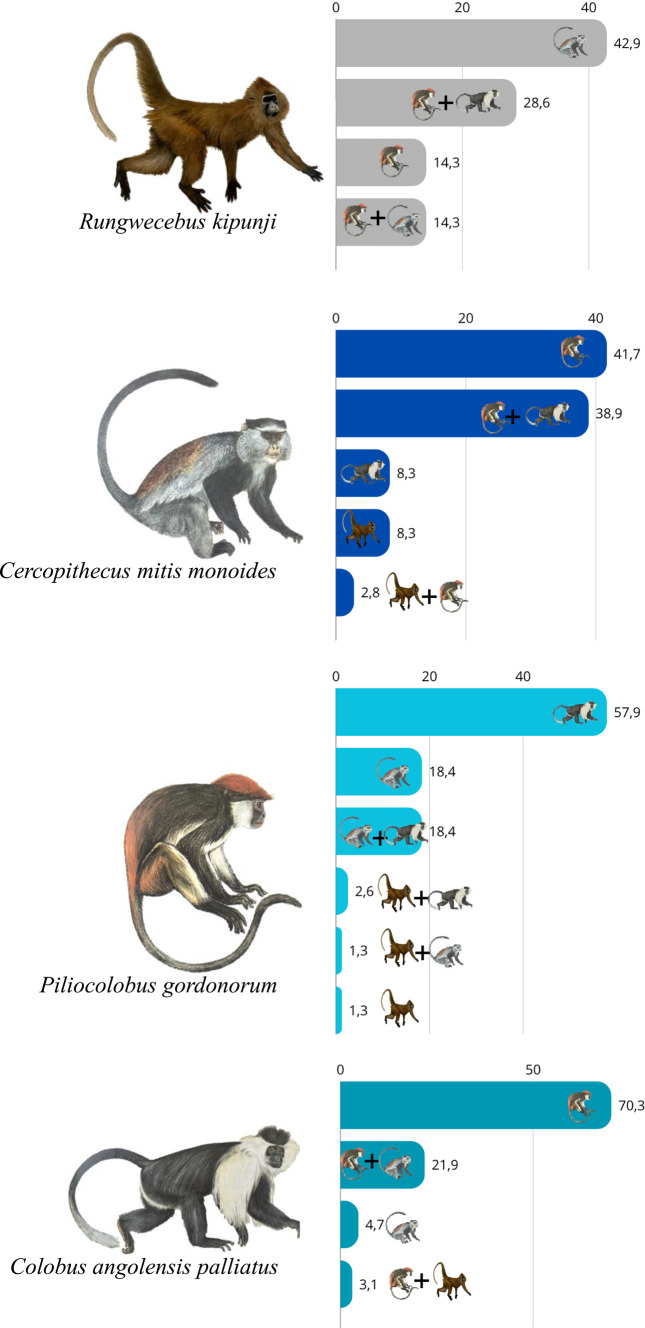
Proportion of polyspecific associations among Udzungwa primates. Bar plots show, the percentage of associations involving one or more primate species for each of the species: kipunji (*Rungwecebus kipunji*), Tanzania Sykes’ monkey Sykes (*Cercopithecus mitis monoides*), Udzungwa red colobus (*Piliocolobus gordonorum*), and Peters’ Angola colobus (*Colobus angolensis palliates*). Data are combined across all survey years. Primate drawings by Jonathan Kingdon (Butynski et al. 2013)

Polyspecific associations were a common feature among the four primate species observed, although their frequency varied among species. When data from all years were combined, Udzungwa red colobus formed polyspecific associations in 58.02% of observations, whereas Tanzania Sykes’ monkeys were more frequently observed alone, with only 32.43% of observations involving polyspecific associations. Kipunji and Peter’s Angola colobus showed intermediate values, being observed in polyspecific associations in 43.75% and 50% of cases, respectively (Fig. 4). The diversity of polyspecific associations varied among the four primate species, likely reflecting differences in social flexibility. Udzungwa red colobus was the most versatile, participating in up to seven different interspecific combinations out of eight possible (including solitary behavior), Tanzania Sykes’ monkey showed moderate flexibility with six possible combinations, while kipunji and Peter’s Angola colobus reached a maximum of five combinations each (Fig. 5).

**Fig. 6:**
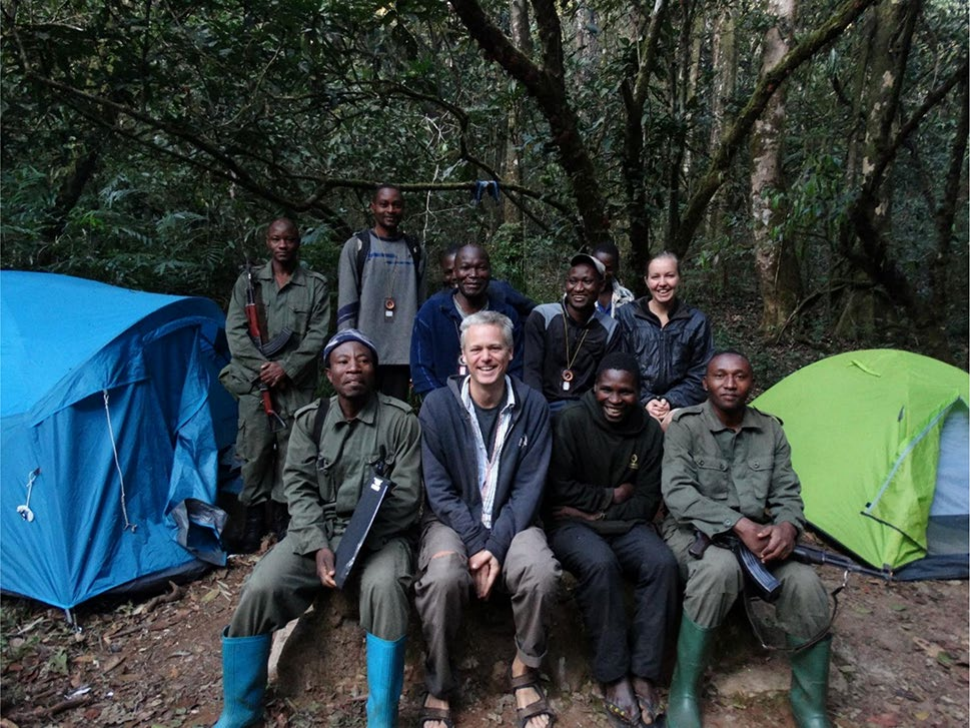
Field team during the 2016 census. The photograph shows the full field team, including Aloyce Mwakisoma (front row, first on left), to whose memory this study is dedicated. Aloyce, a highly respected research assistant from Tanzania’s Udzungwa Mountains, made a fundamental contribution to data collection and field activities. Born and raised in the Udzungwas, he was renowned for his exceptional knowledge of local flora and fauna and widely recognized by colleagues as a local expert whose Indigenous Knowledge substantially informed biodiversity research and conservation efforts. Aloyce tragically passed away in October 2025

All four species showed a marked tendency to associate with one or two other species, while associations including all four species were never recorded. Kipunji most often formed species dyads (57.1% of observations), primarily with Tanzania Sykes’ monkey (42.9%) and less frequently with Udzungwa red colobus (14.3%), while triads accounted for the remaining 42.9% of sightings. Udzungwa red colobus individuals were involved in dyads in 77.6% of the observed polyspecific associations, mostly with Peter’s Angola colobus (57.9%) and Tanzania Sykes’ monkey (18.4%), and only occasionally with kipunji (1.3%). The remaining 22.4% of cases consisted of three-species groups, predominantly including Udzungwa red colobus, Peter’s Angola colobus and Tanzania Sykes’ monkey (18.4%), whereas associations involving Udzungwa red colobus, kipunji and Peter’s Angola colobus or Tanzania Sykes’ monkey were less common (3.9%). Tanzania Sykes’ monkey associated primarily with Udzungwa red colobus (41.7%), and less often with Peter’s Angola colobus (8.3%) or kipunji (8.3%). Triads were also frequent, mostly comprising Peter’s Angola colobus, Udzungwa red colobus, and Tanzania Sykes’ monkey (38.9%). Peter’s Angola colobus were observed in association with another species in 75% of cases, mainly with Udzungwa red colobus (70.3%) or Tanzania Sykes’ monkey (4.7%). Three-species groups involving Peter’s Angola colobus most often included Udzungwa red colobus and Tanzania Sykes’ monkey (21.9%), whereas those with kipunji were rare (3.1%) Fig. 6.

## Discussion

Our study confirms that the kipunji population in the Udzungwa Mountains is extremely small and range-restricted, with density that appears lower than that reported for the Southern Highlands (Davenport et al. 2022), while the relative abundance of other sympatric primates was in the range of those found in other undisturbed Udzungwa forests (Cavada et al. 2016).

## Kipunji encounter rate and demography

Given that survey effort deployed in the Udzungwa Mountains has been broadly comparable across the four survey years, the few groups recorded likely represent the entire remaining population in the Udzungwa Mountains. Our approach did not consider imperfect detection as we could not use inferential methods to estimate abundance, and hence we cannot exclude that additional groups might have been missed; however, it still allowed us to compare relative abundance across years and provide a minimum but coherent total count of groups and individuals. The results suggest that despite fluctuations in the group counts (3—6) no clear temporal trend is detectable, hence we suggest that population abundance has not changed dramatically over the past two decades. Our minimum, inferred abundance of approximately 100 individuals is consistent with the estimate of 75 individuals based on both the baseline sweep census and additional, focal group counts in 2006 (Davenport et al. 2008). In contrast, the MCP encompassing all sightings (10.42 km^2^) is less than half the area of 25 km^2^ indicated by Davenport (2019), but closely matches the range estimated by TJ (13 km^2^; unpubl. data 2017). It is critical to observe that several surveys have been conducted across the remaining portion of Ndundulu forest and even stretching into the contiguous Luhomero forest in UMNP and into other forest blocks of the landscape, reporting no presence of kipunji (Jones et al. 2019; TJ and FR unpubl.). Therefore, we are confident that ours is a precise measure of current population range, reflecting a residual distribution in the most interior suitable habitat, which coincides with the lower elevation zone of Ndundulu forest, represented by closed, old-growth montane forest (Marshall et al. 2015).

Although based on minimum counts and related to a fraction of individuals within the observed groups, data on group sizes and age–sex compositions were broadly consistent across surveys and aligned with those recorded from the Southern Highlands, suggesting that social structure may have remained stable over time and across populations. Social groups in the Udzungwa Mountains were moderately large (averaging 17 individuals) and indicate a demographic pattern similar to that reported from the Southern Highlands (Davenport et al. 2022). However, a substantial fraction of individuals in the Udzungwa Mountains (41.8%) were classified as *unknown*, which most likely belonged to subadults and juveniles — the same age classes reported to represent 52.9% of the population in the Southern Highlands (compared to only 12.7% in our dataset) and collected thanks to extensive, focal observational studies conducted in this area (Davenport et al. 2022). Therefore, the discrepancies in group composition between populations are likely due to detectability issues and differences in effort deployed, as younger age classes in kipunji tend to be more elusive and thus less easily observed during transect surveys (Davenport et al. 2008).

## Udzungwa primates’ encounter rates

The encounter rates of both Udzungwa red colobus and Peter’s Angola colobus are broadly consistent with previous estimates from other forested areas of the Udzungwa Mountains. For Udzungwa red colobus, rates reported in Mwanihana (0.45), Matundu (0.47), and Magombera (0.62) align with our long-term data (0.32–0.70 groups per km between 2006 and 2024), while for Peter’s Angola colobus, previous values in Magombera (0.55), Matundu (0.42), and Mwanihana (0.42) closely match our observations (0.47 in 2006, 0.37 in 2013, 0.50 in 2016, and 0.42 in 2024). By contrast, encounter rates for both colobines are markedly lower in the Uzungwa Scarp (0.08 groups per km), where targeted hunting has drastically impacted both colobine monkeys (Araldi et al. 2014; Barelli et al. 2023).

Tanzania Sykes’ monkeys show a slightly different pattern, with encounter rates in Ndundulu initially lower (0.11) than in other Udzungwa forests (0.38–0.69). Interestingly, however, their numbers appear to have increased across the censuses (0.55 in 2024), reaching levels comparable to those elsewhere (Araldi et al. 2014; Barelli et al. 2023). This increase may be related to a decline in kipunji numbers, reducing interspecific competition and allowing Tanzania Sykes’ monkey to exploit a broader range of resources and habitats within the forest (see also below). This pattern may be consistent with a density compensation phenomenon, whereby more generalist and smaller-bodied guenon species expand their spatial occurrence as larger-bodied and/or more specialized primates decline or become less detectable (Peres and Dolman 2000).

## Kipunji rarity and polyspecific associations

The restricted range and low density of kipunji in Udzungwa is puzzling. Biogeographic and paleo-climatic processes have determined a drastic contraction in the past and the separation into two populations; indeed Ndundulu forest has been suggested to have acted as a refuge for ancient lineages (e.g., Stanley et al. 2005) given the presence of other species endemic to Ndundulu and the nearby Nyumbanitu forest, such as *Congosorex phillipsorum* and *Xenoperdix udzungwensis* (Dinesen et al. 1994; Stanley et al. 2005), and the grey-faced sengi found only in Ndundulu-Luhomero and Mwanihana in the eastern UMNP (Rovero et al. 2008). Kipunji in Udzungwa may not have been able to disperse to other forests following the ancient range contraction, reflecting the inferred long-term isolation between the two populations and reduced genetic diversity (Zinner et al. 2018). While reasons underlying their current low abundance relative to the Southern Highlands population also remain unknown, our results can help shed light.

The high-quality habitat—characterized by high plant diversity and the availability of preferred food items (Marshall et al. 2015)—where kipunji occurs suggests that additional factors may have been at play. Evidence from the Southern Highlands shows that the species can tolerate disturbed habitats relatively well (Bracebridge et al. 2011), suggesting that anthropogenic disturbance alone is unlikely to explain its low density in the Udzungwa Mountains. Moreover, no direct hunting of kipunji has been recently documented in the area (STEP, unpubl. patrol data), nor do we have data confirming that hunting occurred in the past. Recent habitat loss appears minimal compared to nearby forests within the Udzungwa range (e.g., Barelli et al. 2023; Oberosler et al. 2020). Interspecific interactions are another candidate factor influencing kipunji’s population dynamics and its current resilience (Davenport and Butynski 2013). Such interactions likely have a dual role depending on the identity and ecological overlap of the species involved.

Our results revealed that polyspecific associations were more frequently observed between kipunji and, in order, Tanzania Sykes’ monkey, Peter’s Angola colobus, and Udzungwa red colobus. However, Tanzania Sykes’ monkeys are also the species that resulted to associate the least (38%), and that increased in number and distribution over the 19 years covered by the surveys initiated in 2006. When they did form associations, these were highly selective, occurring 89% of the time with the two folivorous colobines. Their territorial and cohesive social structures may limit flexibility for interspecific interactions, favouring associations with species that minimize dietary competition, such as the strictly folivorous colobines. These results suggest that Tanzania Sykes’ monkeys engage in polyspecific associations selectively, balancing the benefits of mixed-species interactions with the advantages of relying on abundant conspecific groups, which may explain the fraction of associations with kipunji despite the higher dietary overlap. Moreover, the observed mean group size of Tanzania Sykes’ monkeys in Udzungwa (4.74 ± 2.20 individuals) is notably low relative to other *Cercopithecus mitis* populations studied elsewhere (e.g., 14–33 individuals in Ngogo, Kakamega, and Ethiopia: Mekonnen et al. 2020; Frogge et al. 2022), which may drive them to form polyspecific associations as a strategy for enhanced protection and access to food. While these considerations remain speculative in lack of additional ecological data, they suggest that the Tanzania Sykes’ monkeys may function as a behaviourally dominant and ecologically flexible species in the guild of arboreal primates. This could influence kipunji, which shares similar dietary preferences (details below) despite being relatively larger, in ways consistent with patterns observed in Uzungwa scarp from long-term monitoring (Barelli et al. 2023; Rovero et al. 2012).

Kipunji were observed primarily in dyads (57.2%), most often with Tanzania Sykes’ monkey (42.9%) and, less frequently, with Udzungwa red colobus, while triads occurred in a smaller proportion of encounters. Following the reasoning above, the associations between kipunji and Tanzania Sykes’ monkey likely reflect a combination of ecological overlap and behavioural advantages, rather than being solely driven by dietary factors. Although both species are broadly omnivorous–frugivorous, Tanzania Sykes’ monkeys tend to be more frugivorous and opportunistic (Coleman and Hill 2015), whereas kipunji is reported to consume a higher proportion of leaves, bark, and pith, particularly when fruit availability declines (Davenport et al. 2006). This partial trophic differentiation may reduce direct feeding competition while allowing the two species to benefit from each other’s ecological needs, such as locating fruiting trees or detecting potential threats (Chapman and Chapman 2000). Indeed, besides feeding, predator avoidance represents an important selective pressure favouring polyspecific associations (Gautier-Hion et al. 1983; Terborgh 1990). Ndundulu forest is inhabited by both crowned eagles (*Stephanoaetus coronatus*) and leopards (*Panthera pardus*), which pose significant predation risks (TJ and FR unpubl.). Mixed-species groups may thus experience increased vigilance and earlier predator detection, as documented for primate associations elsewhere (Cords 1990a, b; Stojan-Dolar and Heymann 2010). Tanzania Sykes’ monkeys are highly vocal and responsive to potential danger, enhancing group-level awareness. Interestingly, kipunji associate more frequently with Tanzania Sykes’ monkey than with Peter’s Angola colobus, a difference that can be attributed to lower ecological overlap and limited behavioral compatibility with the latter. Colobines are primarily folivorous, use higher canopy strata, and are relatively silent, providing fewer foraging and anti-predator benefits. Taken together, these observations suggest that for kipunji, associating with Tanzania Sykes’ monkey might represent a context-dependent mutualism in which moderate dietary overlap, spatial proximity, and enhanced predator detection outweigh the potential costs of interspecific competition. Yet, this association appears to be less prioritized by Tanzania Sykes’ monkeys themselves for the reasons explained above.

Concerning the other species, Udzungwa red colobus seemed the most socially flexible, engaging in a higher proportion of associations (58% of the time they were observed in association with another primate species) and participating in a wider range of interspecific combinations. This pattern may reflect their high degree of dietary specialization: as mainly folivorous primates (Struhsaker 2010), feeding predominantly on young leaves, they may experience limited direct competition with other species more frugivorous or omnivorous (i.e., kipunji and Tanzania Sykes’ monkey). Additionally, their tendency to form relatively cohesive and predictable groups might make them attractive association partners, providing reliable cues on habitat safety or resource availability to other species (i.e., Chapman and Chapman 2000).

All in all, our results suggest that polyspecific associations may confer selective advantages, such as enhanced vigilance (e.g., Cords 1990a, b; Stojan-Dolar and Heymann 2010) or improved foraging efficiency (Chapman and Chapman 2000), particularly for the rare kipunji, while also reflecting interspecific compatibility and social preferences (Daoudi-Simison et al. 2024; Heymann and Buchanan-Smith 2000). Associations involving all four species were never recorded, indicating potential limits to social integration across species or differences in temporal and spatial activity patterns, as suggested for Amazonian forest primates (Buchanan-Smith 1999; Haugaasen and Peres 2009). Unfortunately, no direct data on dietary preferences, vigilance, or predation risk are available to further elucidate the mechanisms underlying these patterns.

In conclusion, our study confirms the restricted range and low abundance of kipunji in Udzungwa, highlighting the vulnerability of this population to stochastic events and emerging threats, such as climate change and the potential expansion of human activities along forest edges (Davenport 2019). Maintaining habitat integrity through continued and enhanced protection, monitoring demographic trends and boosting focal socio-ecological studies are critical actions forward. Furthermore, we acknowledge that any increase in research effort must be carefully balanced against potential risks associated with greater human presence. The remoteness and inhospitability of the Ndundulu Forest may indeed offer the remaining kipunji a degree of protection, and therefore the costs and benefits of intensified research activities—including the risk of human-to-kipunji disease transmission—should be thoughtfully considered when planning future studies.

This study provides updated and novel insights into the ecology and social behaviour of kipunji in the Udzungwas, showing that even if occurring in apparently suitable habitat, the population persistence is at elevated risk. Urgent attention and targeted conservation strategies, with the exclusion of any translocation from the Southern Highlands population due to their well-established genetic differentiation, are essential to safeguard this flagship and endangered population.

## Data Availability

The data supporting this study are not publicly available, but can be provided by the corresponding author upon reasonable request.
